# 7-Phenyl-7*H*-dinaphtho­[2,1-*b*:1′,2′-*d*]phosphole 7-oxide

**DOI:** 10.1107/S1600536811038177

**Published:** 2011-09-30

**Authors:** Lan Yao, Chang-Qiu Zhao

**Affiliations:** a College of Chemistry and Chemical Engineering, Liaocheng University, Shandong 252059, People’s Republic of China

## Abstract

In the title compound, C_26_H_17_OP, the naphthyl ring systems are bent away from each other [dihedral angle = 30.81 (8)°]. In the crystal, weak inter­molecular C—H⋯O inter­actions link the mol­ecules into helical chains along the 2_1_ screw axis.

## Related literature

For applications of organo­phospho­rus compounds, see: Antczak & Montchamp (2008 ▶); Yan & Zhang (2005 ▶). For related structures, see: Tani *et al.* (1994 ▶); Gowda *et al.* (2010 ▶).
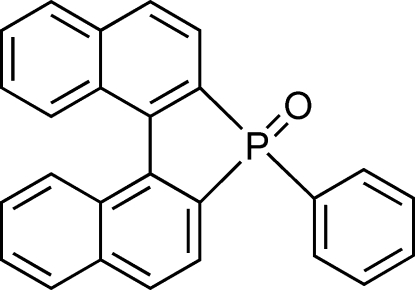


## Experimental

### 

#### Crystal data

C_26_H_17_OP
*M* *_r_* = 376.37
Monoclinic, 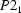

*a* = 10.9637 (12) Å
*b* = 8.0627 (8) Å
*c* = 11.0352 (13) Å
β = 94.746 (1)°
*V* = 972.13 (18) Å^3^
*Z* = 2
Mo *K*α radiation
μ = 0.16 mm^−1^
*T* = 298 K
0.26 × 0.17 × 0.13 mm

#### Data collection

Bruker SMART 1000 CCD area-detector diffractometer
Absorption correction: multi-scan (*SADABS*; Sheldrick, 1996 ▶) *T*_min_ = 0.961, *T*_max_ = 0.980
4947 measured reflections
3317 independent reflections
2431 reflections with *I* > 2σ(*I*)
*R*_int_ = 0.027

#### Refinement

*R*[*F* ^2^ > 2σ(*F* ^2^)] = 0.042
*wR*(*F* ^2^) = 0.065
*S* = 1.03
3317 reflections
253 parameters
1 restraint
H-atom parameters constrained
Δρ_max_ = 0.25 e Å^−3^
Δρ_min_ = −0.21 e Å^−3^
Absolute structure: Flack (1983 ▶); 1455 Friedel pairs
Flack parameter: 0.00 (9)

### 

Data collection: *SMART* (Bruker, 2007 ▶); cell refinement: *SAINT* (Bruker, 2007 ▶); data reduction: *SAINT*; program(s) used to solve structure: *SHELXS97* (Sheldrick, 2008 ▶); program(s) used to refine structure: *SHELXL97* (Sheldrick, 2008 ▶); molecular graphics: *SHELXTL* (Sheldrick, 2008 ▶); software used to prepare material for publication: *SHELXTL*.

## Supplementary Material

Crystal structure: contains datablock(s) I, global. DOI: 10.1107/S1600536811038177/cv5145sup1.cif

Supplementary material file. DOI: 10.1107/S1600536811038177/cv5145Isup3.cml

Additional supplementary materials:  crystallographic information; 3D view; checkCIF report

## Figures and Tables

**Table 1:** Hydrogen-bond geometry (Å, °)

*D*—H⋯*A*	*D*—H	H⋯*A*	*D*⋯*A*	*D*—H⋯*A*
C25—H25⋯O1^i^	0.93	2.47	3.229 (4)	139

Symmetry code: (i) 
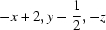
.

**Fig. 1.**
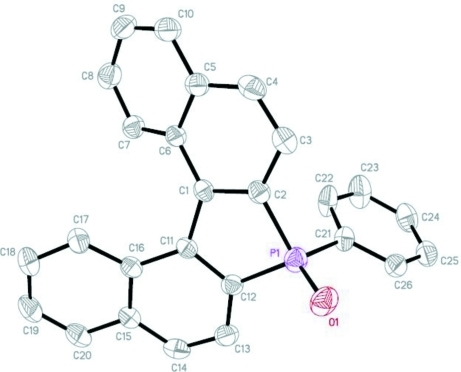
The molecular structure of the title compound, showing the atomic numbering scheme with 50% probability displacement ellipsoids. H atoms have been omitted for clarity.

## supplementary crystallographic information

### Comment

Organophosphorus compounds are important in various applications (Antczak *et al.*, 2008), including asymmetric catalysis (Yan & Zhang, 2005). In our search for new organophosphorus compounds, we obtained the title compound (I) unintentionally.

The molecule of (I) (Fig. 1) is chiral because of the bent naphthyl rings. All bond lengths and angles are normal and comparable with those observed in the related compounds (Tani *et al.*, 1994; Gowda *et al.*, 2010). Weak intermolecular C—H···O hydrogen bonds (Table 1) link molecules into helical chains along screw axis 2_1_.

### Experimental

*n*-BuLi(0.2 mmol of a 1.6*M* hexane solution) was added dropwise to a solution of (*R*)-2,2'-dibromo-1,l'-binaphthyl (0.1 mmol) in dry ether(5 ml) at -30°C. After stirring for 3 h to form (*R*)-2,2-Dilithio-l,l'-binaphthalene, it was added to dichlorophenylphosphine(0.1 mmol) at -80°C for 0.5 h, then the mixture was stirred from -80°C to room temperature overnight. After washing with water, the resulting solution was purified by silica gel plate to afford optically pure product 7-phenyl-7*H*-benzo[*e*]naphtho[2,1-*b*]phosphindole. The crystal suitable for X-ray diffraction was obtained from recrystallization with acetone, during this process the product was oxidized to form the title compound.

### Refinement

All H atoms attached to C atoms were fixed geometrically and treated as riding, with C—H = 0.93 - 0.98 Å, with *U*_iso_(H) = 1.5 *U*_eq_(methyl) and *U*_iso_(H) = 1.2 *U*_eq_(C) for all other H atoms.

### Crystal data

**Table:**

C_26_H_17_OP	*F*(000) = 392
*M**_r_* = 376.37	*D*_x_ = 1.286 Mg m^−^^3^
Monoclinic, *P*2_1_	Mo *K*α radiation, λ = 0.71073 Å
Hall symbol: P 2yb	Cell parameters from 1467 reflections
*a* = 10.9637 (12) Å	θ = 2.5–25.9°
*b* = 8.0627 (8) Å	µ = 0.16 mm^−^^1^
*c* = 11.0352 (13) Å	*T* = 298 K
β = 94.746 (1)°	Block, colourless
*V* = 972.13 (18) Å^3^	0.26 × 0.17 × 0.13 mm
*Z* = 2	

### Data collection

**Table:**

Bruker SMART 1000 CCD area-detector diffractometer	3317 independent reflections
Radiation source: fine-focus sealed tube	2431 reflections with *I* > 2σ(*I*)
graphite	*R*_int_ = 0.027
phi and ω scans	θ_max_ = 25.0°, θ_min_ = 2.5°
Absorption correction: multi-scan (*SADABS*; Sheldrick, 1996)	*h* = −8→13
*T*_min_ = 0.961, *T*_max_ = 0.980	*k* = −9→9
4947 measured reflections	*l* = −11→13

### Refinement

**Table:**

Refinement on *F*^2^	Secondary atom site location: difference Fourier map
Least-squares matrix: full	Hydrogen site location: inferred from neighbouring sites
*R*[*F*^2^ > 2σ(*F*^2^)] = 0.042	H-atom parameters constrained
*wR*(*F*^2^) = 0.065	*w* = 1/[σ^2^(*F*_o_^2^) + (0.0113*P*)^2^] where *P* = (*F*_o_^2^ + 2*F*_c_^2^)/3
*S* = 1.03	(Δ/σ)_max_ < 0.001
3317 reflections	Δρ_max_ = 0.25 e Å^−^^3^
253 parameters	Δρ_min_ = −0.21 e Å^−^^3^
1 restraint	Absolute structure: Flack (1983); 1455 Friedel pairs
Primary atom site location: structure-invariant direct methods	Flack parameter: 0.00 (9)

### Special details

**Table:**

Geometry. All e.s.d.'s (except the e.s.d. in the dihedral angle between two l.s. planes) are estimated using the full covariance matrix. The cell e.s.d.'s are taken into account individually in the estimation of e.s.d.'s in distances, angles and torsion angles; correlations between e.s.d.'s in cell parameters are only used when they are defined by crystal symmetry. An approximate (isotropic) treatment of cell e.s.d.'s is used for estimating e.s.d.'s involving l.s. planes.
Refinement. Refinement of *F*^2^ against ALL reflections. The weighted *R*-factor *wR* and goodness of fit *S* are based on *F*^2^, conventional *R*-factors *R* are based on *F*, with *F* set to zero for negative *F*^2^. The threshold expression of *F*^2^ > σ(*F*^2^) is used only for calculating *R*-factors(gt) *etc*. and is not relevant to the choice of reflections for refinement. *R*-factors based on *F*^2^ are statistically about twice as large as those based on *F*, and *R*- factors based on ALL data will be even larger.

### Fractional atomic coordinates and isotropic or equivalent isotropic displacement parameters (Å^2^)

**Table:**

	*x*	*y*	*z*	*U*_iso_*/*U*_eq_	
O1	0.90190 (19)	0.5234 (2)	0.13528 (15)	0.0670 (6)	
P1	0.81611 (7)	0.41973 (10)	0.20150 (6)	0.0522 (2)	
C1	0.6484 (3)	0.4877 (3)	0.3524 (2)	0.0407 (7)	
C2	0.6672 (3)	0.4991 (3)	0.2295 (2)	0.0452 (7)	
C3	0.5726 (3)	0.5462 (4)	0.1420 (2)	0.0563 (8)	
H3	0.5873	0.5517	0.0603	0.068*	
C4	0.4601 (3)	0.5835 (3)	0.1773 (3)	0.0599 (8)	
H4	0.4009	0.6272	0.1210	0.072*	
C5	0.4318 (3)	0.5569 (3)	0.2986 (3)	0.0519 (8)	
C6	0.5254 (3)	0.4972 (3)	0.3874 (2)	0.0437 (7)	
C7	0.4870 (3)	0.4388 (4)	0.5001 (2)	0.0530 (7)	
H7	0.5438	0.3892	0.5561	0.064*	
C8	0.3681 (3)	0.4543 (4)	0.5274 (3)	0.0653 (9)	
H8	0.3448	0.4142	0.6010	0.078*	
C9	0.2806 (3)	0.5308 (4)	0.4447 (3)	0.0709 (10)	
H9	0.2018	0.5496	0.4670	0.085*	
C10	0.3107 (3)	0.5773 (4)	0.3326 (3)	0.0670 (9)	
H10	0.2512	0.6229	0.2775	0.080*	
C11	0.7644 (2)	0.4480 (3)	0.4292 (2)	0.0423 (7)	
C12	0.8558 (2)	0.3895 (3)	0.3616 (2)	0.0447 (7)	
C13	0.9672 (3)	0.3282 (4)	0.4170 (3)	0.0574 (8)	
H13	1.0266	0.2876	0.3693	0.069*	
C14	0.9880 (3)	0.3285 (4)	0.5413 (3)	0.0590 (9)	
H14	1.0580	0.2786	0.5781	0.071*	
C15	0.9036 (3)	0.4043 (4)	0.6133 (2)	0.0499 (7)	
C16	0.7915 (3)	0.4729 (3)	0.5589 (2)	0.0452 (7)	
C17	0.7202 (3)	0.5734 (3)	0.6322 (2)	0.0552 (8)	
H17	0.6510	0.6268	0.5968	0.066*	
C18	0.7516 (3)	0.5927 (4)	0.7538 (3)	0.0667 (9)	
H18	0.7039	0.6589	0.8002	0.080*	
C19	0.8567 (3)	0.5122 (4)	0.8097 (3)	0.0711 (10)	
H19	0.8749	0.5208	0.8933	0.085*	
C20	0.9316 (3)	0.4221 (4)	0.7416 (2)	0.0644 (8)	
H20	1.0012	0.3720	0.7790	0.077*	
C21	0.7879 (3)	0.2185 (3)	0.1327 (2)	0.0501 (8)	
C22	0.6998 (3)	0.1138 (4)	0.1745 (2)	0.0703 (10)	
H22	0.6498	0.1511	0.2329	0.084*	
C23	0.6864 (3)	−0.0466 (5)	0.1290 (3)	0.0860 (11)	
H23	0.6280	−0.1174	0.1572	0.103*	
C24	0.7609 (3)	−0.0997 (4)	0.0415 (2)	0.0720 (10)	
H24	0.7527	−0.2075	0.0121	0.086*	
C25	0.8467 (3)	0.0030 (4)	−0.0029 (2)	0.0640 (9)	
H25	0.8951	−0.0340	−0.0627	0.077*	
C26	0.8599 (3)	0.1633 (4)	0.0430 (2)	0.0541 (8)	
H26	0.9175	0.2341	0.0135	0.065*	

### Atomic displacement parameters (Å^2^)

**Table:**

	*U*^11^	*U*^22^	*U*^33^	*U*^12^	*U*^13^	*U*^23^
O1	0.0700 (14)	0.0669 (14)	0.0672 (13)	−0.0132 (12)	0.0243 (11)	0.0051 (11)
P1	0.0559 (5)	0.0518 (5)	0.0497 (4)	−0.0046 (5)	0.0092 (4)	−0.0004 (4)
C1	0.0473 (19)	0.0344 (15)	0.0398 (16)	−0.0025 (13)	−0.0004 (13)	−0.0029 (12)
C2	0.0487 (19)	0.0425 (17)	0.0440 (16)	−0.0046 (14)	0.0014 (14)	−0.0004 (13)
C3	0.064 (2)	0.0561 (19)	0.0476 (18)	−0.0035 (18)	−0.0030 (17)	0.0003 (15)
C4	0.058 (2)	0.056 (2)	0.062 (2)	0.0044 (18)	−0.0135 (17)	0.0009 (16)
C5	0.049 (2)	0.0460 (18)	0.060 (2)	0.0014 (16)	−0.0009 (17)	−0.0092 (15)
C6	0.0422 (18)	0.0421 (17)	0.0463 (16)	−0.0029 (14)	0.0003 (14)	−0.0081 (13)
C7	0.052 (2)	0.0500 (18)	0.0570 (17)	−0.0023 (18)	0.0072 (14)	−0.0078 (16)
C8	0.065 (2)	0.061 (2)	0.071 (2)	−0.007 (2)	0.0170 (18)	−0.0120 (18)
C9	0.051 (2)	0.071 (2)	0.092 (3)	0.002 (2)	0.014 (2)	−0.026 (2)
C10	0.053 (2)	0.061 (2)	0.085 (2)	0.0067 (18)	−0.0076 (19)	−0.0237 (19)
C11	0.0437 (17)	0.0383 (17)	0.0442 (15)	−0.0042 (14)	−0.0016 (13)	−0.0024 (13)
C12	0.0407 (18)	0.0426 (19)	0.0505 (16)	−0.0013 (15)	0.0026 (14)	−0.0036 (14)
C13	0.047 (2)	0.0593 (19)	0.067 (2)	−0.0005 (17)	0.0077 (17)	−0.0057 (16)
C14	0.044 (2)	0.059 (2)	0.072 (2)	−0.0013 (17)	−0.0088 (18)	0.0041 (17)
C15	0.0506 (19)	0.0494 (18)	0.0488 (17)	−0.0048 (18)	−0.0022 (15)	−0.0006 (16)
C16	0.0475 (19)	0.0418 (18)	0.0452 (16)	−0.0038 (14)	−0.0017 (14)	0.0019 (13)
C17	0.063 (2)	0.054 (2)	0.0480 (18)	0.0026 (17)	0.0000 (16)	−0.0047 (15)
C18	0.085 (3)	0.072 (2)	0.0428 (18)	−0.005 (2)	−0.0008 (17)	−0.0093 (16)
C19	0.083 (3)	0.084 (3)	0.0433 (19)	−0.012 (2)	−0.0124 (18)	−0.0026 (17)
C20	0.063 (2)	0.072 (2)	0.0548 (19)	−0.006 (2)	−0.0160 (16)	0.005 (2)
C21	0.060 (2)	0.050 (2)	0.0409 (17)	0.0031 (16)	0.0077 (15)	0.0005 (13)
C22	0.105 (3)	0.056 (2)	0.0545 (19)	−0.019 (2)	0.0333 (19)	−0.0145 (16)
C23	0.129 (3)	0.067 (3)	0.065 (2)	−0.030 (2)	0.026 (2)	−0.0043 (18)
C24	0.099 (3)	0.058 (2)	0.057 (2)	0.000 (2)	−0.0018 (19)	−0.0132 (18)
C25	0.070 (2)	0.072 (2)	0.0487 (19)	0.019 (2)	−0.0009 (16)	−0.0050 (17)
C26	0.053 (2)	0.061 (2)	0.0479 (18)	0.0046 (17)	0.0022 (15)	0.0022 (16)

### Geometric parameters (Å, °)

**Table:**

O1—P1	1.4937 (18)	C13—C14	1.372 (3)
P1—C12	1.801 (2)	C13—H13	0.9300
P1—C2	1.803 (3)	C14—C15	1.408 (3)
P1—C21	1.807 (3)	C14—H14	0.9300
C1—C2	1.391 (3)	C15—C20	1.431 (3)
C1—C6	1.435 (3)	C15—C16	1.434 (3)
C1—C11	1.503 (3)	C16—C17	1.424 (3)
C2—C3	1.410 (3)	C17—C18	1.366 (3)
C3—C4	1.357 (4)	C17—H17	0.9300
C3—H3	0.9300	C18—C19	1.418 (4)
C4—C5	1.415 (3)	C18—H18	0.9300
C4—H4	0.9300	C19—C20	1.366 (4)
C5—C10	1.419 (4)	C19—H19	0.9300
C5—C6	1.443 (3)	C20—H20	0.9300
C6—C7	1.425 (3)	C21—C26	1.389 (3)
C7—C8	1.368 (3)	C21—C22	1.390 (4)
C7—H7	0.9300	C22—C23	1.390 (4)
C8—C9	1.410 (4)	C22—H22	0.9300
C8—H8	0.9300	C23—C24	1.384 (4)
C9—C10	1.359 (4)	C23—H23	0.9300
C9—H9	0.9300	C24—C25	1.373 (4)
C10—H10	0.9300	C24—H24	0.9300
C11—C12	1.381 (3)	C25—C26	1.391 (4)
C11—C16	1.451 (3)	C25—H25	0.9300
C12—C13	1.409 (3)	C26—H26	0.9300
			
O1—P1—C12	116.43 (12)	C14—C13—C12	119.9 (3)
O1—P1—C2	120.06 (12)	C14—C13—H13	120.0
C12—P1—C2	91.62 (12)	C12—C13—H13	120.0
O1—P1—C21	112.92 (12)	C13—C14—C15	120.2 (3)
C12—P1—C21	108.04 (12)	C13—C14—H14	119.9
C2—P1—C21	105.37 (13)	C15—C14—H14	119.9
C2—C1—C6	118.6 (2)	C14—C15—C20	120.3 (3)
C2—C1—C11	112.1 (3)	C14—C15—C16	120.8 (2)
C6—C1—C11	129.0 (2)	C20—C15—C16	118.8 (3)
C1—C2—C3	121.7 (3)	C17—C16—C15	118.2 (2)
C1—C2—P1	110.7 (2)	C17—C16—C11	124.2 (2)
C3—C2—P1	127.1 (2)	C15—C16—C11	117.3 (2)
C4—C3—C2	119.8 (3)	C18—C17—C16	121.1 (3)
C4—C3—H3	120.1	C18—C17—H17	119.4
C2—C3—H3	120.1	C16—C17—H17	119.4
C3—C4—C5	120.9 (3)	C17—C18—C19	120.3 (3)
C3—C4—H4	119.6	C17—C18—H18	119.8
C5—C4—H4	119.6	C19—C18—H18	119.8
C4—C5—C10	121.1 (3)	C20—C19—C18	120.5 (3)
C4—C5—C6	119.6 (3)	C20—C19—H19	119.8
C10—C5—C6	119.2 (3)	C18—C19—H19	119.8
C7—C6—C1	124.7 (3)	C19—C20—C15	120.6 (3)
C7—C6—C5	117.3 (3)	C19—C20—H20	119.7
C1—C6—C5	117.7 (2)	C15—C20—H20	119.7
C8—C7—C6	121.2 (3)	C26—C21—C22	119.6 (3)
C8—C7—H7	119.4	C26—C21—P1	120.0 (2)
C6—C7—H7	119.4	C22—C21—P1	120.3 (2)
C7—C8—C9	120.5 (3)	C23—C22—C21	120.1 (3)
C7—C8—H8	119.8	C23—C22—H22	120.0
C9—C8—H8	119.8	C21—C22—H22	120.0
C10—C9—C8	120.4 (3)	C22—C23—C24	119.2 (3)
C10—C9—H9	119.8	C22—C23—H23	120.4
C8—C9—H9	119.8	C24—C23—H23	120.4
C9—C10—C5	120.8 (3)	C25—C24—C23	121.6 (3)
C9—C10—H10	119.6	C25—C24—H24	119.2
C5—C10—H10	119.6	C23—C24—H24	119.2
C12—C11—C16	118.8 (2)	C24—C25—C26	118.9 (3)
C12—C11—C1	112.7 (2)	C24—C25—H25	120.5
C16—C11—C1	128.4 (2)	C26—C25—H25	120.5
C11—C12—C13	121.8 (2)	C21—C26—C25	120.6 (3)
C11—C12—P1	110.9 (2)	C21—C26—H26	119.7
C13—C12—P1	127.1 (2)	C25—C26—H26	119.7
			
C6—C1—C2—C3	10.5 (4)	C2—P1—C12—C11	−4.2 (2)
C11—C1—C2—C3	−175.7 (2)	C21—P1—C12—C11	−110.9 (2)
C6—C1—C2—P1	−161.64 (19)	O1—P1—C12—C13	−54.1 (3)
C11—C1—C2—P1	12.2 (3)	C2—P1—C12—C13	−179.1 (3)
O1—P1—C2—C1	−126.99 (18)	C21—P1—C12—C13	74.2 (3)
C12—P1—C2—C1	−4.9 (2)	C11—C12—C13—C14	−1.2 (4)
C21—P1—C2—C1	104.28 (19)	P1—C12—C13—C14	173.2 (2)
O1—P1—C2—C3	61.4 (3)	C12—C13—C14—C15	−6.1 (4)
C12—P1—C2—C3	−176.5 (2)	C13—C14—C15—C20	−172.7 (3)
C21—P1—C2—C3	−67.3 (3)	C13—C14—C15—C16	3.9 (4)
C1—C2—C3—C4	0.7 (4)	C14—C15—C16—C17	−169.0 (3)
P1—C2—C3—C4	171.5 (2)	C20—C15—C16—C17	7.6 (4)
C2—C3—C4—C5	−7.7 (4)	C14—C15—C16—C11	5.3 (4)
C3—C4—C5—C10	−172.6 (3)	C20—C15—C16—C11	−178.1 (3)
C3—C4—C5—C6	3.1 (4)	C12—C11—C16—C17	161.6 (3)
C2—C1—C6—C7	159.9 (2)	C1—C11—C16—C17	−14.2 (4)
C11—C1—C6—C7	−12.7 (4)	C12—C11—C16—C15	−12.2 (4)
C2—C1—C6—C5	−14.5 (3)	C1—C11—C16—C15	172.0 (3)
C11—C1—C6—C5	172.9 (2)	C15—C16—C17—C18	−5.6 (4)
C4—C5—C6—C7	−166.8 (3)	C11—C16—C17—C18	−179.4 (3)
C10—C5—C6—C7	8.9 (4)	C16—C17—C18—C19	0.0 (4)
C4—C5—C6—C1	8.0 (4)	C17—C18—C19—C20	3.7 (5)
C10—C5—C6—C1	−176.2 (3)	C18—C19—C20—C15	−1.5 (5)
C1—C6—C7—C8	179.1 (3)	C14—C15—C20—C19	172.4 (3)
C5—C6—C7—C8	−6.4 (4)	C16—C15—C20—C19	−4.2 (5)
C6—C7—C8—C9	−0.8 (5)	O1—P1—C21—C26	11.5 (3)
C7—C8—C9—C10	5.7 (5)	C12—P1—C21—C26	−118.7 (2)
C8—C9—C10—C5	−2.9 (5)	C2—P1—C21—C26	144.4 (2)
C4—C5—C10—C9	171.3 (3)	O1—P1—C21—C22	−172.7 (2)
C6—C5—C10—C9	−4.4 (4)	C12—P1—C21—C22	57.1 (3)
C2—C1—C11—C12	−15.8 (3)	C2—P1—C21—C22	−39.8 (3)
C6—C1—C11—C12	157.2 (2)	C26—C21—C22—C23	1.8 (5)
C2—C1—C11—C16	160.2 (2)	P1—C21—C22—C23	−174.0 (3)
C6—C1—C11—C16	−26.8 (4)	C21—C22—C23—C24	−0.5 (5)
C16—C11—C12—C13	10.5 (4)	C22—C23—C24—C25	−1.0 (5)
C1—C11—C12—C13	−173.1 (2)	C23—C24—C25—C26	1.1 (5)
C16—C11—C12—P1	−164.76 (18)	C22—C21—C26—C25	−1.6 (4)
C1—C11—C12—P1	11.7 (3)	P1—C21—C26—C25	174.1 (2)
O1—P1—C12—C11	120.84 (19)	C24—C25—C26—C21	0.2 (4)

### Hydrogen-bond geometry (Å, °)

**Table:**

*D*—H···*A*	*D*—H	H···*A*	*D*···*A*	*D*—H···*A*
C25—H25···O1^i^	0.93	2.47	3.229 (4)	139.

Symmetry codes: (i) −*x*+2, *y*−1/2, −*z*.
